# Effectiveness and safety profile of S-1-based chemotherapy compared with capecitabine-based chemotherapy for advanced gastric and colorectal cancer: A meta-analysis

**DOI:** 10.3892/etm.2014.1576

**Published:** 2014-02-24

**Authors:** JIA-XIANG YE, AI-QUN LIU, LIAN-YING GE, SHAO-ZHANG ZHOU, ZHONG-GUO LIANG

**Affiliations:** 1 Department of Medical Oncology, The Cancer Institute, Affiliated Tumor Hospital of Guangxi Medical University, Nanning, Guangxi 530021, P.R. China; 2 Department of Radiation Oncology, The Cancer Institute, Affiliated Tumor Hospital of Guangxi Medical University, Nanning, Guangxi 530021, P.R. China

**Keywords:** S-1, capecitabine, advanced gastric cancer, advanced colorectal cancer, meta-analysis

## Abstract

The aim of the present analysis was to compare the efficacy and safety profile of S-1-based chemotherapy (SBCT) versus capecitabine-based chemotherapy (CBCT) for advanced gastric cancer (AGC) and advanced colorectal cancer (ACRC). A meta-analysis was performed, which included eligible randomized controlled trials (RCTs) that were identified using RevMan 5.1.0 software. A total of 1,064 patients from 11 RCTs, comprising of 527 patients in the SBCT group and 537 patients in the CBCT group, were included in the analysis. For AGC, the meta-analysis of overall survival (OS) [hazard ratio (HR), 0.98; 95% confidence interval (CI), 0.85–1.12], time to progression (HR, 0.95; 95% CI, 0.80–1.12) and overall response rate (ORR) [odds ratio (OR), 1.06; 95% CI, 0.72–1.55] of patients in the SBCT group indicated no statistical significance when compared with those in the CBCT group. Furthermore, for ACRC, a pooled analysis demonstrated no significant difference between the SBCT and CBCT groups (OS: HR, 0.82; 95% CI, 0.61–1.10; progression-free survival: HR, 0.79; 95% CI=0.60–1.04; ORR: OR, 1.27; 95% CI, 0.91–1.78). The statistically significant differences identified in the overall meta-analysis indicated a low incidence of grade 3–4 hand-foot-syndrome (OR, 0.15; 95% CI, 0.06–0.36) in the SBCT group; however no statistically significant difference was observed in the incidence of grade 3–4 anemia, thrombocytopenia, leucopenia, neutropenia, diarrhea, stomatitis or nausea/vomiting. The SBCT treatment exhibited similar efficacy and an approximately equivalent safety profile compared with the CBCT treatment and was an alternative to CBCT for patients with AGC or ACRC; however, further investigation is required to provide confirmation.

## Introduction

Regardless of advances in their diagnosis and treatment, gastric and colorectal cancer remain a common cause of cancer-related mortality worldwide (1,2). Acquiring curative therapy by surgery or radiotherapy is complex for patients with advanced gastric cancer (AGC) or advanced colorectal cancer (ACRC); therefore, systemic chemotherapy is considered to be the primary effective treatment.

Traditional continuous infusion of 5-fluorouracil (5-FU) in combination with folinic acid has been the primary chemotherapeutic treatment for AGC and ACRC. However, continuous infusion requires an indwelling central venous catheter; thus, there is an increased risk of infection and thrombosis, in addition to the requirement for regular hospital visits. Therefore, administration of the traditional 5-FU-based chemotherapy (FBCT) is time-consuming, uncomfortable and inconvenient for patients.

Recently, oral fluoropyrimidines have been developed as a substitution for 5-FU infusion therapy. Capecitabine is an oral fluoropyrimidine that was designed to simulate a continuous intravenous infusion of 5-FU (3) and has been approved for the treatment of patients with AGC and ACRC. S-1 is another treatment, which is a combination of three pharmacological compounds: Tegafur, gimeracil and oteracil potassium at a molar ratio of 1:0.4:1, respectively (4), which has been identified as a suitable alternative to 5-FU for the treatment of patients with ACRC or AGC (5,6).

As the efficacy and safety of oral fluoropyrimidines, capecitabine and S-1, have been confirmed, it is necessary to identify which treatment exhibits a greater efficacy and safety profile for AGC and ACRC patients. To date, there have been a series of trials comparing S-1 with capecitabine as part of a mono or combined therapeutic treatment (7–17); however, a single study may not be sufficient to comprehensively assess their efficacy and safety. Moreover, to the best of our knowledge, a meta-analysis of S-1-based chemotherapy (SBCT) versus capecitabine-based chemotherapy (CBCT) for ACRC or AGC has not been conducted. Therefore, the present meta-analysis of eligible studies was performed to compare the two treatment approaches and evaluate their clinical efficacy and safety in patients with AGC and ACRC.

## Materials and methods

### Literature search

A comprehensive search was conducted using PubMed (http://www.ncbi.nlm.nih.gov/pubmed), Embase (https://www.embase.com), the Cochrane Library (http://www.thecochranelibrary.com) and the Chinese Biological Medical Database (http://www.sinomed.ac.cn/zh/) to identify randomized controlled trials (RCTs) from inception of the databases to August 31^st^ 2013. Various combinations of different terms, such as ‘gastric cancer’, ‘colorectal cancer’, ‘capecitabine’, ‘S-1’, ‘randomized’ and their synonyms served as search terms. The following search strategy of Embase was used: i) ‘gastric neoplasm’: ab, ti OR ‘gastric cancer’: ab, ti OR ‘gastric carcinoma’: ab, ti OR ‘stomach cancer’: ab, ti OR ‘cancer of stomach’: ab, ti OR ‘stomach neoplasm’: ab, ti OR ‘stomach cancer’/exp OR ‘colorectal carcinoma’: ab, ti OR ‘colorectal cancer’: ab, ti OR ‘colorectal neoplasm’: ab, ti OR ‘rectal cancer’: ab, ti OR ‘colon cancer’: ab, ti OR ‘intestinal cancer’: ab, ti OR ‘colorectal carcinoma’/exp. ii) ‘tegafur gimeracil oteracil potassium’: ab, ti OR ‘S-1’: ab, ti OR ‘TS-1’: ab, ti OR ‘gimeracil plus oteracil potassium plus tegafur’/exp. iii) ‘capecitabine’: ab, ti OR ‘xeloda’: ab, ti OR ‘capecitabine’/exp. iv) random*: ab, ti OR ‘randomized controlled trial’: ab, ti OR ‘controlled clinical trial’: ab, ti OR ‘randomized controlled trial’/exp OR ‘randomized controlled trial (topic)’/exp. v) #1 AND #2 AND #3 AND #4. This strategy was applied to the search of other databases.

In addition, all of the abstracts from the American Society of Clinical Oncology conferences (http://meetinglibrary.asco.org/) that were held between 2003 and 2013 were searched to identify the relevant RCTs and references that were cited in the identified articles were searched manually. The search was conducted without any restriction on language.

### Inclusion and exclusion criteria

The inclusion and exclusion criteria were delineated prior to commencement of the literature search. The eligible studies were included in the present meta-analysis if they met all of the following criteria: i) Participants were patients with histologically confirmed, advanced, recurrent or metastatic colorectal or gastric cancer and did not present with severe, basic diseases which may affect the treatment effect of patients (including cardiovascular and cerebrovascular diseases); ii) only RCTs were considered; iii) trials compared SBCT with CBCT, particularly mono- or combined therapy of S-1 versus capecitabine, without confusion resulting from the administration of additional drugs or interventions (for example, experimental and control arms exhibited differences between S-1 and capecitabine components alone within a combination therapy). Accordingly, the following exclusion criteria were used: i) Cross-over studies; ii) non-randomized or single-arm phase II trials; iii) any review, letter, case report or comment; iv) for repeated published articles or the same study of a different follow-up period, the study with the strictest methodology and most complete data was selected and the other excluded.

### Data extraction

The essential data was independently extracted from the eligible studies by two investigators and any discrepancies were resolved by a consensus between the two. Information was collected from each study as follows: The first author’s name, publishing year, country/region of origin, study design, characteristics of the participants, interventions conducted and outcomes. When the hazard ratio (HR) of overall survival (OS), progression-free survival (PFS) and time to progression (TTP) could not be directly extracted from the original reports, they were extracted from Kaplan-Meier curves as reported by Tierney *et al* (18).

### Quality assessment of the included studies

The quality of the eligible studies was assessed by two investigators independently and any disagreements were resolved by a third investigator. According to the Cochrane Collaboration’s tool for assessing risk of bias of RCTs (5.1.0) (19), the following criteria were used to appraise the RCTs included in full texts: Random sequence generation, allocation concealment, binding of participants and personnel, binding of outcome assessments, incomplete outcome data, selective reporting and other bias. In all cases, high, low or unclear risk was used to evaluate the risk of bias; when there was insufficient detail included in the study, the judgment was that the risk of bias was unclear.

### Statistical analysis

Statistical analysis of the HR and 95% confidence interval (CI) for OS, PFS and TTP, in addition to the odds ratio (OR) and 95% CI for the overall response rate (ORR) and grade 3 or 4 adverse events (AEs) were calculated using RevMan 5.1.0 software. The ORR was defined as the sum of the partial and complete response rates according to the Response Evaluation Criteria in Solid Tumors (20). A fixed-effects model was initially used and the Q test and I^2^ statistical test were subsequently performed to assess the heterogeneity between studies; P<0.1 was considered to indicate a statistically significant difference. When there was heterogeneity across the trials, sensitivity analysis or a randomized-effect model was applied to overcome this shortcoming, and the process of sensitivity analysis excluding the study firstly according to different inclusion criteria and then re-analyzing the remaining studies. For the results mentioned above (not including the heterogeneity test): P<0.05 was considered to indicate a statistically significant difference. OR>1 indicated a favorable outcome in the S-1-based group; however, it indicated a greater level of toxicity and a HR>1 demonstrated a greater number of fatalities or progression with S-1-based regimens for OS or TTP and PFS, respectively.

## Results

### Literature search

**Figure 1:**
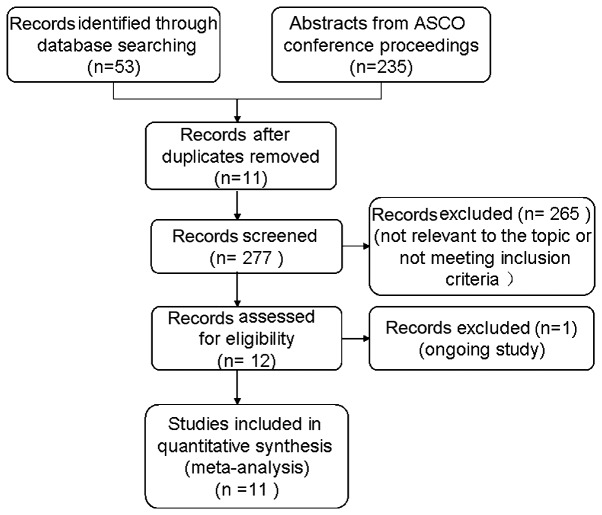
Flow chart displaying the process of study selection for the present meta-analysis. ASCO, American Society of Clinical Oncology.

The search strategy yielded 288 records, of these, 11 duplicates were eliminated and 265 articles were excluded due to irrelevancy or failing to meet the inclusion criteria, which was determined by a review of the titles and abstracts. Further evaluation of the remaining 12 studies revealed that one study was ongoing (21). Thus, 11 studies (7–17) qualified for inclusion in the present meta-analysis (Fig. 1). Table I displays the characteristics of the 11 individual studies with respect to author, year, country, demographic data, interventions and the study outcomes.

### Quality of eligible studies

The present meta-analysis included 11 RCTs and all of the studies included the term ‘random’, however, only three studies (7,8,14) specifically reported the methods that were utilized for random sequence generation. Furthermore, only two studies (7,9) adequately reported the reliability-determined allocation concealment. Three trials (7,9,10) were open-labeled and the other studies did not state whether a blind method was adopted; however, these were unlikely to affect the quality assessment results. One trial was an abstract and included insufficient information regarding the outcome data, selective reporting and other bias (10), additional trials satisfied the criteria for complete outcome data and did not include selective reporting or other bias (Table II).

### Tumor response

**Figure 2:**
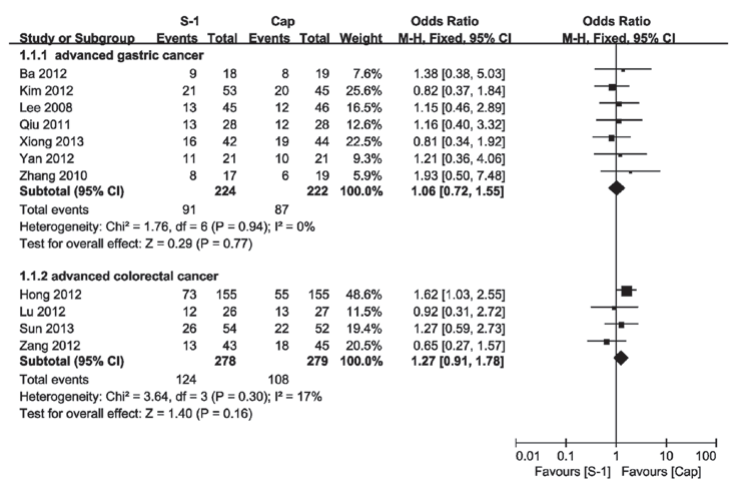
Forest plot of the odds ratio of the overall response rate. CI, confidence interval.

All of the included studies provided the information on ORR and the pooled OR of ORR for AGC of the fixed-effect model was 1.06 (95% CI, 0.72 and 1.55) with no heterogeneity (P=0.94, I^2^=0%; Fig. 2). For ACRC, there was no heterogeneity identified across the trials (P=0.30, I^2^=17%) and the pooled OR of ORR using the fixed-effect model was 1.27 (95% CI, 0.91 and 1.78). The OR indicated that there was no significant difference between the SBCT and CBCT group.

### TTP or PFS

**Figure 3:**
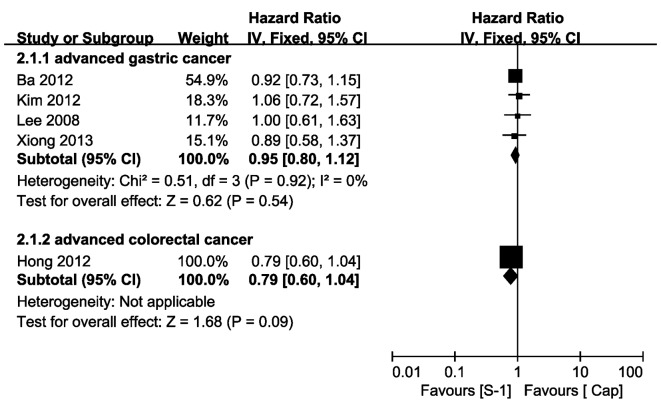
Forest plot of the hazard ratio of time to progression or progression-free survival. CI, confidence interval.

Regarding AGC, four trials provided TTP information (8,9,11,15), of which the TTP HR of the trial reported by Xiong *et al* (15) was extracted from the Kaplan-Meier curves that were presented in the study. The pooled HR of TTP indicated that there was no significant difference between the SBCT and CBCT group (HR=0.95, 95% CI, 0.80–1.12) and the pooled HR was performed using a fixed-effect model, with no heterogeneity observed (P=0.92, I^2^=0%). Concerning ACRC, no trials provided the HR of TTP and only one trial provided PFS data (7); moreover, the HR of PFS identified no significant difference between the SBCT and CBCT group (HR=0.79, 95% CI, 0.60–1.04; Fig. 3).

### OS data

**Figure 4:**
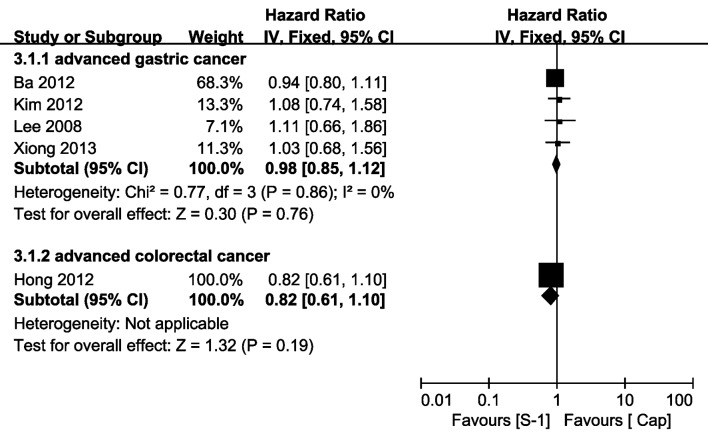
Forest plot of the hazard ratio of overall survival. CI, confidence interval.

Five of the 12 trials provided OS data (7–9,11,15), of which the OS HR of the trial, reported by Xiong *et al* (15)*,* was extracted from the Kaplan-Meier curves that were presented in the study. Regarding AGC, there was no significant heterogeneity observed between the studies (P=0.86, I^2^=0%), the pooled HR of OS obtained using a fixed-effect model showed no significant difference between the SBCT and CBCT group, yielding a HR of 0.98 (95% CI, 0.85–1.12). Concerning ACRC, only one trial provided the HR of OS (HR=0.82, 95% CI, 0.61–1.10) (7), which demonstrated that there was no significant difference between the two groups (Fig. 4).

### Safety profile

#### Neutropenia in hematologic toxicities

A meta-analysis of seven trials (7–11,15,16) regarding grade 3–4 neutropenia, which included 398 patients in the SBCT group and 399 patients in the CBCT group, identified no significant difference between the two groups (OR=1.17, 95%CI, 0.81–1.70), with significant heterogeneity observed across the trials (P=0.02, I^2^=61%) and as the sensitivity analysis did not identify the source of the heterogeneity, a random-effect model was applied. The result of the random-effect model showed that there was no significant difference between the two groups (OR=0.79, 95% CI, 0.37–1.69).

#### Leucopenia

A meta-analysis of seven trials (7–9,12–14,17) regarding grade 3–4 leucopenia, which included 401 patients in the SBCT group and 400 patients in the CBCT group, showed no significant difference between the two groups (OR=0.91, 95% CI, 0.37–2.24) and there was no significant heterogeneity identified across the trials (P=0.72, I^2^=0%).

#### Thrombocytopenia

Nine trials (7–12,15–17) reported grade 3–4 thrombocytopenia from the assessment of 889 participants (SBCT, n=441; CBCT, n=445). The pooled analysis showed no significant difference between the two groups (OR=1.34, 95% CI, 0.89–2.02) and significant heterogeneity was demonstrated across the trials (P=0.01, I^2^=62%). As the heterogeneity could not be eliminated by conducting a sensitivity analysis, a random-effect model was performed (OR=0.85, 95% CI, 0.36–1.97).

#### Anemia

Data concerning anemia was available from nine trials (7–13,15,16), which included 906 participants (SBCT, n=452; CBCT, n=454) in the meta-analysis. The pooled OR of the nine trials indicated no significant difference between the two groups (OR=1.61, 95% CI, 0.93–2.80) and there was no heterogeneity noted between the studies (P=0.88, I^2^=0.0%).

#### Hand-foot syndrome (HFS) in non-hematologic toxicities

Ten trials (7–13,15–17) reported grade 3–4 HFS (SBCT, n=469; CBCT, n=473). The pooled OR of grade 3–4 HFS (OR=0.15, 95% CI, 0.06–0.36) showed that there was a statistically significant difference between the SBCT and CBCT groups, with no significant heterogeneity observed across the trials (P=0.99, I^2^=0%), which indicated that grade 3–4 HFS was less likely to occur in SBCT patients.

#### Diarrhea and nausea/vomiting

Ten studies (7–9,11–17) that included 482 patients in the SBCT group and 484 patients in the CBCT group, provided information on cases of grade 3–4 diarrhea and nausea/vomiting. The meta-analysis of the 10 trials showed no significant difference between the two groups (diarrhea: OR=1.00, 95% CI, 0.56–1.78; nausea/vomiting: OR=0.83, 95% CI, 0.48–1.43) and there was no heterogeneity identified.

#### Stomatitis

Eight trials (7,9,11,13–17) reported grade 3–4 stomatitis, these trials included 391 patients in the SBCT group and 393 patients in the CBCT group. The meta-analysis of the fixed-effects model indicated no significant difference between the two groups (OR=1.23, 95% CI, 0.37–4.12; heterogeneity: P=0.88, I^2^=0%).

The results of the grade 3 and 4 AE analyses are displayed in Table III.

## Discussion

To the best of our knowledge, this was the first meta-analysis to evaluate the efficacy and safety profile of SBCT versus CBCT for AGC or ACRC. A total of 1,064 patients from 11 RCTs, including 527 patients in the SBCT group and 537 patients in the CBCT group were analyzed. On the basis of intention-to-treat analysis, with respect to ORR, TTP and OS, the present meta-analysis showed no significant differences between the SBCT and CBCT group for AGC, which suggested that SBCT was not inferior to CBCT for patients with AGC. With regards to ACRC, the pooled analysis indicated that there was no significant difference between the SBCT and CBCT groups concerning the ORR, PFS and OS, which indicated that SBCT exhibited similar efficacy to CBCT for patients with ACRC.

With regard to the safety profile, although the present meta-analysis indicated that grade 3–4 HFS was less likely to occur in SBCT patients (OR=0.15, 95% CI, 0.06–0.36), grade 3–4 toxicities, such as anemia, leucopenia, neutropenia, thrombocytopenia, nausea/vomiting, diarrhea and stomatitis were identified to be similarly prevalent between the two groups. The types of toxicities were deemed to be manageable, predictable and tolerable. Therefore, compared with CBCT, SBCT exhibited an approximately equivalent safety profile for patients with AGC and ACRC.

Owing to the significant heterogeneity observed between grade 3–4 neutropenia and grade 3–4 thrombocytopenia, a sensitivity analysis was performed; however, the factors contributing to the heterogeneity could not be identified, therefore, they may be associated with variations in age, performance status of patients, dose and the regimen of therapy between the trials. Thus, a random-effects model was applied to compensate for this and the conclusion did not alter relative to the result of the fixed-effects model, which confirmed the reliability of our outcome.

Of note, the patient population included in the present meta-analysis was markedly heterogeneous, i.e. the patients exhibited metastatic colorectal or advanced gastric cancer. Thus, subgroup analysis by tumor type was performed on ORR, TTP and OS; the findings of which indicated that the non-inferiority of SBCT versus CBCT could be applied to all of the patient subgroups that were considered within the present analysis. However, as the type of toxicity was not specific to a certain type of tumor for the same drug and AGC and ACRC are gastrointestinal cancers, a greater significance was determined from the results of the overall meta-analysis of grade 3–4 AEs for all of the patients, rather than the results from the subgroup population analysis.

In 2009, a meta-analysis of individual patient data was conducted by Okines *et al* (22) and demonstrated that OS was superior in patients with AGC that were treated with CBCT compared with the patients with AGC that were treated with FBCT (HR=0.87, 95% CI, 0.77–0.98). Similarly, a meta-analysis was performed by Huang *et al* (23) comparing the use of FBCT, which identified a significant OS benefit in favor of SBCT for patients with AGC (HR=0.87, 95% CI, 0.79–0.96). The data from these two meta-analyses indirectly showed that SBCT exhibited a similar efficacy to CBCT for AGC patients, which was in line with the present study.

In addition, S-1 and capecitabine possess different optimal doses, efficacy and safety among patients that are from different regions. Based on the metabolic pathway, capecitabine exhibited no obvious racial difference concerning the dose and efficacy regardless of a lower grade 3–4 gastrointestinal toxicity when compared with FBCT in Asian patients (24). However, as the polymorphic variants of the liver enzyme gene, cytochrome P450 (CYP) 2A6, which converts tegafur to 5-FU, were more frequent in Asians than in Caucasians, there were certain racial differences identified regarding S-1 tolerance, which results in varying optimal doses of S-1 for different races (25). The study by Kong *et al* showed that the CYP2A6 genotype was associated with the treatment efficacy of SBCT in AGC patients (26) and it may result in certain racial differences in S-1 efficacy. In 2009, the updated results of a randomized phase III study, conducted by Fuse *et al* (27), demonstrated that S-1 was associated with an increased OS compared with 5-FU in Japanese patients with AGC (HR=0.83, 95% CI, 0.68–1.00, P=0.02). However, in 2010 the findings of a RCT conducted by Ajani *et al* (28) showed that SBCT did not prolong the OS of Western patients with AGC when compared with FBCT (HR=0.92, 95% CI, 0.80–1.05, P=0.20).

There were certain limitations in the present meta-analysis. As all of the studies included were from Asia, the present results require confirmation from studies conducted on patients from a Western population. Moreover, the quantity of included studies was small and there may have been publication bias. The quality of the studies was not considered to be high, with just three studies (7,8,14) reporting the specific methods of random sequence generation and two studies (7,9) reporting with adequate reliability the method by which the allocation concealment was determined; thus, a greater number of RCTs with improved methodologies are required to update the present study. Furthermore, information was not obtained from individual patients for each trial, which would have resulted in a more comprehensive analysis and, finally, heterogeneous results were included.

In conclusion, the present meta-analysis indicated that SBCT exhibited a comparable efficacy and approximately equivalent safety profile in patients with AGC and ACRC compared with CBCT; therefore, it was considered to be an effective alternative to CBCT. However, further investigation, which includes large-scale prospective studies with an adequate methodological quality and legitimate control measures that account for possible confounding factors are required to confirm or update the present study, particularly including patients from a Western population.

## Figures and Tables

**Table I:** Predominant characteristics of the studies included in the present meta-analysis.

Study (ref.)	Country/tumor type	No. of patients^a^	PS (score)	SBCT regimen	CBCT regimen	Outcome measures
Kim *et al* 2012 (8)	Korea/AGC	65/64	0–2	S-1: 80 mg/m^2^ d1–14, oxaliplatin: 130 mg/m^2^ d1, q3w	Capecitabine: 2,000 mg/m^2^ d1–14 oxaliplatin: 130 mg/m^2^ d1, q3w	ORR, OS, TTP, QOL, toxicities
Lee *et al* 2008 (9)	Korea/AGC	45/46	0–2	S-1: 40–60 mg, bid, according to body surface area, d1–28, q6w	Capecitabine: 1,250 mg/m^2^, bid, d1–14, q3w	ORR, TTP, OS, toxicities
Xiong *et al* 2013 (15)	China/AGC	42/44	>70^b^	Docetaxel: 25 mg/m^2^ d1 d8 d15, S-1: 80 mg/m^2^, d1–14, q4w	Docetaxel: 25 mg/m^2^ d1 d8 d15, capecitabine: 1250 mg/m^2^ d1–14, q4w	ORR, MST, toxicities
Zhang *et al* 2010 (17)	China/AGC	17/19	≥60^b^	S-1: 80 mg/m^2^, d1–28, q6w	Capecitabine: 2500 mg/m^2^ d1–14, q3w	ORR, toxicities
Ba *et al* 2012 (11)	China/AGC	18/19	0–1	S-1: 80 mg/m^2^ d1–14, cisplatin: 75 mg/m^2^ d1, q3w	Capecitabine: 2000 mg/m^2^ d1–14; cisplatin: 75 mg/m^2^ d1, q3w	ORR, TTP, OS, toxicities
Yan *et al* 2012 (16)	China/AGC	21/21	-	S-1: 40 mg/m^2^, bid, d1–14, cisplatin: 75 mg/m^2^ d1, q3w	Capecitabine: 1000 mg/m^2^, bid, d1–14; cisplatin: 75 mg/m^2^ d1, q3w	ORR, toxicities
Qiu *et al* 2011 (13)	China/AGC	28/28	≥60^b^	S-1: 40 mg/m^2^, bid, d1–28, q5w	Capecitabine: 1,250 mg/m^2^, bid, d1–14, q3w	ORR, toxicities
Hong *et al* 2012 (7)	Korea/ACRC	168/172	0–2	S-1: 40 mg/m^2^, bid, d1–14, oxaliplatin: 130 mg/m^2^ d1, q3w	Capecitabine: 1000 mg/m^2^, bid, d1–14, oxaliplatin: 130 mg/m^2^ d1, q3w	ORR, TTF, PFS, OS, toxicities
Sun *et al* 2013 (14)	China/ACRC	54/52	>70^b^	S-1: 80 mg/m^2^, d1–14, q3w	Capecitabine: 2000 mg/m^2^, d1–14, q3w	ORR, MST, toxicities
Lu *et al* 2012 (12)	China/ACRC	26//27	0–2	S-1: 80 mg/m^2^ d1–14; oxaliplatin: 130 mg/m^2^ d1, q3w	Capecitabine: 2000 mg/m^2^ d1–14, oxaliplatin: 130 mg/m^2^ d1, q3w	ORR, toxicities
Zang *et al* 2012 (10)	Korea/ACRC	43/45	0–2	S-1: 80 mg/m^2^ d1–14, oxaliplatin: 130 mg/m^2^ d1, q3w	Capecitabine: 2000 mg/m^2^ d1–14, oxaliplatin: 130 mg/m^2^ d1, q3w	ORR, TTP, MST, toxicities

a SBCT/CBCT regimen patients,

b Karnofsky method.

PS, performance status; SBCT, S-1-based chemotherapy; CBCT, capecitabine-based chemotherapy; AGC, advanced gastric cancer; ORR, overall response rate; OS, overall survival; TTP, time to progression; QOL, quality of life; MST, median survival time; PFS, progression-free survival; ACRC, advanced colorectal cancer; TTF, time to treatment failure; d1–14, days 1–14; q3w, every 3 weeks; q4w, every 4 weeks; q6w, every 6 weeks.

**Table II:** Risk of bias for each study.

	Risk of bias						
	
Study (ref.)	A	B	C	D	E	F	G
Kim *et al*, 2012 (8)	Low	Unclear	Low	Low	Low	Low	Low
Lee *et al*, 2008 (9)	Unclear	Low	Low	Low	Low	Low	Low
Xiong *et al*, 2013 (15)	Unclear	Unclear	Low	Low	Low	Low	Low
Zhang *et al*, 2010 (17)	Unclear	Unclear	Low	Low	Low	Low	Low
Ba *et al*, 2012 (11)	Unclear	Unclear	Low	Low	Low	Low	Low
Yan *et al*, 2012 (16)	Unclear	Unclear	Low	Low	Low	Low	Low
Qiu *et al*, 2011 (13)	Unclear	Unclear	Low	Low	Low	Low	Low
Hong *et al*, 2012 (7)	Low	Low	Low	Low	Low	Low	Low
Sun *et al*, 2013 (14)	Low	Unclear	Low	Low	Low	Low	Low
Lu *et al*, 2012 (12)	Unclear	Unclear	Low	Low	Low	Low	Low
Zang *et al*, 2012 (10)	Unclear	Unclear	Low	Low	Unclear	Unclear	Unclear

A, random sequence generation; B, allocation concealment; C, binding of participants and personnel; D, binding of outcome assessment; E, incomplete outcome data; F, selective reporting; G, other bias.

**Table III:** Outcome of the toxicity meta-analysis comparing SBCT with CBCT in advanced gastric and colorectal cancer.

				Heterogenity				
							
Toxicity	Trials	SBCT	CBCT	P-value	I^2^ (%)	OR (95% CI)	Model	References
Grade 3–4 anemia	9	35/452	23/454	0.88	0	1.61 (0.93, 2.80)	Fixed	(7–13,15,16)
Grade 3–4 leucopenia	7	9/401	10/400	0.72	0	0.91 (0.37, 2.24)	Fixed	(7–9,12–14,17)
Grade 3–4 neutropenia	7	72/398	63/399	0.02	61	0.79 (0.37, 1.69)	Random	(7–11,15,16)
Grade 3–4 thrombocytopenia	9	61/441	47/445	0.01	62	0.85 (0.36, 1.97)	Random	(7–12,15–17)
Grade 3–4 diarrhea	10	21/482	21/484	0.42	2	1.00 (0.56, 1.78)	Fixed	(7–9,11–17)
Grade 3–4 nausea/vomiting	10	23/482	28/484	0.69	0	0.83 (0.48,1.43)	Fixed	(7–9,11–17)
Grade 3–4 stomatitis	8	5/391	4/393	0.88	0	1.23 (0.37, 4.12)	Fixed	(7,9,11,13–17)
Grade 3–4 HFS	10	3/469	33/473	0.99	0	0.15 (0.06, 0.36)	Fixed	(7–13,15–17)

HFS, hand-foot syndrome; SBCT, S-1-based chemotherapy; CBCT, capecitabine-based chemotherapy; OR, odds ratio; CI, confidence interval.
